# Talking really does matter: Lay perspectives from older people on talking about suicide in later life

**DOI:** 10.3389/fpsyg.2022.1009503

**Published:** 2022-11-16

**Authors:** Trish Hafford-Letchfield, Jeffrey R. Hanna, Toby J. Ellmers, Susan Rasmussen, Nicola Cogan, Helen Gleeson, Jolie Goodman, Sophie Martin, Patrick Walker, Matthew Quaife

**Affiliations:** ^1^School of Social Work and Social Policy, Faculty of Humanities and Social Sciences, University of Strathclyde, Glasgow, United Kingdom; ^2^Department of Brain Sciences, Faculty of Medicine, Imperial College London, London, United Kingdom; ^3^School of Psychological Sciences and Health, Faculty of Humanities and Social Sciences, University of Strathclyde, Glasgow, United Kingdom; ^4^School of Mental Health and Social Work, School of Education, Middlesex University, London, United Kingdom; ^5^Mental Health Foundation, London, United Kingdom

**Keywords:** aging, suicidal thought, later life, self-harm, social care, health care, mental health, lay perspectives

## Abstract

**Background:**

The cumulative body of research on suicidality in later life describes its unique and complex features in older people when compared with that in other population groups. Yet significant gaps exist in how research informs the further development of suitable interventions. The perspectives of older people are also limited in research findings.

**Aims:**

Therefore, this exploratory study aimed to (1) identify potential barriers and enablers in discussing suicidal thoughts and their expression in later life from the perspectives of lay older people and (2) explore where opportunities might occur in approach, place, relationships, and language with older people to discuss suicidal thoughts and their expression.

**Method:**

We conducted in-depth qualitative individual interviews with 15 people aged 70–89 years. This method helped explore older peoples' own lay perspectives on suicidal thoughts in later life and how these are expressed, and their understanding of where and how people might seek support.

**Results:**

A total of three themes were generated from the dataset: (1) intergenerational and socio-cultural differences in suicide expression, (2) the normalization of suicidal thoughts in later life, and (3) the importance and difficulties of everyday discussion and opportunities to express suicidal thoughts.

**Conclusion:**

Suicidal thoughts and their expression appear commonly and are normalized in later life yet remain taboo and hidden. The participants revealed how such thoughts and behaviors are typically expressed through colloquial or “off-hand” remarks and comments and the importance of authentic listening. The findings highlight the importance of more informal discussions around these topics and how care professionals, practitioners, and providers might frame opportunities for dialogue with people who may want to access support. Further engagement with community-informed participatory research methods in which older people provide their own perspectives and experiences is important in addressing these gaps. There is a need for co-designing in developing screening, assessment, and signposting outside of clinical settings that can be used in everyday caring relationships with people in later life.

## Introduction

Suicidal behavior in later life remains a major public health concern (De Leo, 2022; Laflamme et al., 2022). Despite the decline in the suicide incidence globally, older adults continue to have the highest rates of suicide worldwide (Naghavi, 2019; World Health Organization, 2019). The high rates of suicide observed in later life is thought to be a consequence of the aging population demonstrating more determined and planful suicide acts, coupled with fewer warnings or detection of suicidal intent (Lachman et al., 2015). Prevalence may also be under-estimated and under-recorded due to its unique presentation in later life. Indeed, deaths in older people that result from more passive acts such as the voluntary stopping of eating and drinking (VSED) and suspending/refusing medication are unlikely to be formally investigated as potential suicide deaths or recorded in official statistics (Deuter et al., 2016; Hafford-Letchfield et al., 2020; De Leo, 2022). Unlike other regions of the world, in the United Kingdom, where this study was conducted, suicide is not a criminal act (*Section 2 of the Suicide Act 1961*), and assisted suicide and euthanasia are illegal (UK Parliament, 2015).

There is a cumulative body of theoretical and conceptual research exploring the topic of suicide and aging. This includes extensive reviews of the literature, which have examined, among other themes, the risk factors and characteristics of suicide and self-harm in older adults living both in the community and long-term care (Murphy et al., 2015; Wand et al., 2018; Gleeson et al., 2019; Troya et al., 2019). Reviews have also addressed our understanding of suicide intentions in older adults (Diehl-Schmid et al., 2017) and self-injurious behavior (Mahgoub et al., 2011), as well as the role of social factors in suicidal behavior (Chang et al., 2017). However, gaps remain in relation to how research can inform further development of prevention, intervention, and postvention in suicide expression in later life, given its unique and complex features, compared with suicide expression in other groups (Van Orden and Conwell, 2011). Later life can be a period when people experience dramatic changes in social status and role (Bernier et al., 2020; Hafford-Letchfield et al., 2020). Some researchers have engaged with the interpersonal theory of suicide (Joiner, 2005), which refers to thwarted belongingness and perceived burdensomeness, combined with an acquired capability for suicide (Van Orden et al., 2010). The need for a better understanding of processes leading to suicidal thoughts as separate to suicidal behavior has more recently been informed by the current drivers of suicide research toward the development of ideation-to-action frameworks (Bayliss et al., 2021). One key message is that recognizing triggers that cause older people to “give up” on life can be difficult (Azulai and Walsh, 2015; Hafford-Letchfield et al., 2020). A recent conceptual model described several (so-called “gray area”) suicidal behaviors that might be considered unique to suicide expression in later life (Hafford-Letchfield et al., 2022a). Yet, these expressions are viewed by many as “normal” part of aging, with such behaviors often described as a “rational” response to an age-related decline in physical or mental function (De Leo, 2022). The conceptual model presented by Hafford-Letchfield et al. (2022a) described how an older adult viewing their life as “completed” could lead to the development of a wish to die and subsequently motivate engagement with a range of behaviors that either are self-destructive (e.g., self-neglect and problematic substance use) or hasten death (e.g., voluntary stopping of eating or drinking and refusing necessary medication).

Studies have also shown that many older people who had died by suicide had consulted a medical practitioner in a period close to their death, and that they had commonly presented with somatic or physical health issues (Harwood et al., 2000; Neufeld and O'Rourke, 2009). These physical health issues may mask psychological difficulties including suicidal thought. At the same time, however, people with an existential sense of completed life, or a wish to die, are less likely to be in touch with professionals, particularly clinicians (van Wijngaarden et al., 2019; Hafford-Letchfield et al., 2020). Further qualitative research on age-related factors on the traumatic impact of suicide bereavement (Hybholt et al., 2020) has demonstrated the relationship between grief and the lack of motivation to carry on and adapt to the physical and psychological effects of growing older. These impacts are also known to trigger suicidal thoughts and/or the wish for the hastening of the end of life (Hafford-Letchfield et al., 2022b).

The use of psychometrically sound screening tools is recommended as the best practice for targeting suicidal thoughts, allowing clinicians to effectively engage and uniformly assess those at significant risk (Rudd, 2021). However, the aforementioned “gray area” behaviors presented by older people may lead to missed opportunities for healthcare providers to talk to these individuals about their suicide ideation and behavior (Shah and Erlangsen, 2014), particularly if the person has not received a diagnosis of depression. Gleeson et al.'s (2022) review of screening measures found that none of them actively included older people themselves in their development beyond item development (e.g., Edelstein et al., 2009; Carmel, 2017). Whilst the measures frequently included questions on social connectedness and support, none fully engaged with the theoretical issue of burdensomeness as a factor in determining suicide ideation. Furthermore, there is a question about how far the screening tools developed could be applied in the community and other groups of older people outside of clinical health settings, where the majority of people are living (Gleeson et al., 2022). In summary, the existing research highlights the need to raise more awareness of the breadth of suicide expression in later life and to foster greater sensitivity to how it might present. This understanding will serve to provide greater opportunities to recognize and respond to suicide-related expression and behaviors (Frost and Cowie, 2019). Further engagement with community participatory research methods in which the voices of older people can be heard based on their own perspectives and experiences is important in addressing these gaps. Likewise, moving toward co-design of any tools or models of practice could support practice with their utility and application (Gleeson et al., 2022).

Therefore, this research study aimed to directly explore older people's views and perspectives in response to the following research questions: (1) what are the potential barriers and enablers in discussing suicidal thoughts in later life from the perspectives of lay older people; (2) are there any unique factors associated with suicidal thoughts in later life that could aid recognition; and (3) what opportunities might occur in relation to approach, place, relationships, and language to discuss suicidal thoughts?

## Methods

### Study design

An exploratory design was used to determine and better understand the nature of suicidal thoughts in everyday life experienced by older adults. In-depth one-to-one interviews with lay older people through semi-structured interviews were conducted to gather information from key informants with personal experiences, attitudes, perceptions, and beliefs related to the key topics (DeJonckheere and Vaughn, 2019).

### Ethics

All participants were from the United Kingdom, lived in their own homes in the community, and provided informed consent before participation. The study was approved by the local Ethics Committee (Ref: UEC21/67). Conducting research associated with suicide ideation and behavior can be challenging for participants and researchers, and careful consideration was given to minimize harm and, where possible, to maximize benefits to participants. Most of the researchers in our research team were registered professionals in social work, psychology, and mental health, and we paid attention to relational, embodied, and reflexive practice during the process of actual interview. The protocol included training for two team members from an approved provider on “talking about suicide” (Scottish Association of Mental Health), which was then cascaded to the team. This training was used to inform the protocol on how to respond and follow up with any participants who expressed suicide ideation or who had expressed suicide loss (see also Hafford-Letchfield et al., 2022b). A resource leaflet was developed signposting to organizations that provided support, and this was sent to all the participants by e-mail or post immediately after the interview. After 1 week of interview, all participants received a handwritten thankyou card and were reminded again of the resources provided and the importance of self-care.

For the participants, talking about suicide and aging can be sensitive, potentially stigmatizing and distressing (Jovicic and McPherson, 2020). The interview topics were designed to allow the participants to say as much as or as little as possible about their own experiences by framing questions in the third person and using open language, for example, “How would you describe?,” “What is your understanding of?,” and “What do you think would help?” (Naughton-Doe et al., 2022). The progression and timing of questions considered any potential for interview fatigue. The interview closed with a debriefing question “how did you find the interview today?”, and participants were invited to contact author 2 if they wanted any more information after the interview had ended.

For the research team members, the protocol built in capacity for debriefing after interviews and self-care (SAMH, 2020). All team members were encouraged to share a one side briefing of key reflective points with colleagues following each interview to support debriefing. The team had access to a clinical psychologist outside of research supervision.

### Recruitment and sampling

As the team was interested in lay perspectives, we recruited from the public *via* purposive and opportunity sampling. Lay people were targeted as we aimed to capture a broad range of insights and opinions from older people, irrespective of whether they had any direct or personal experience of suicidal thoughts and behaviors. Secondly, our aims encompass understanding how everyday interactions encourage more open conversations about suicidal thoughts. No prior personal experience in the topic was required to participate. The team particularly targeted people aged 70 years and older, from a diverse range of backgrounds as these tend to be less represented in suicide research (Wiktorsson et al., 2016).

A poster describing the research and its purpose was drawn up and circulated both electronically and in a paper form. Recruitment information was disseminated through Twitter, e-mail, and by post to colleagues, acquaintances, and networks in the third sector and local public venues. The participants were offered a £20 gift voucher in recognition of the time they gave in participation. Recruitment took place over 4 months in 2021. Anyone who made contact was provided with a participant information sheet and invited to ask any questions. Subject to giving formal consent, the participants were invited to a one-to-one in-depth interview. The team faced several challenges in recruitment, which is not unusual in suicide research (Lakeman and Fitzgerald, 2009). For example, one community organization, working with people in later life, did not want to put the call for participants in their regular newsletter. They said that they only wanted to “promote positive news”, given that people had been through a difficult time during the COVID-19 lockdown. Some contacts said that they did not want to pass the information on to people they knew as they anticipated that people would find the topic too upsetting to talk about, despite reassurances about the design and approval of the study. This was an important reference for the team in relation to the way in which society talks about and/or silences suicide and further highlighted the challenges in providing opportunities for older people's voices to be heard in suicide research.

### Participants

A total of 15 participants aged 70 years and older were recruited to participate in the research (male = 10/15).

### Interview data collection

Pre-interview participants were asked to complete a short online demographic questionnaire to capture their key characteristics. The interview topic guide comprised open-ended questions informed by the research questions and literature review and were framed to facilitate sensitive conversations and engagement, which included topics such as people's immediate thoughts and reflections on what is meant by suicidal thoughts in later life, what might trigger them, how they might be expressed, and why people talk about it or not. We also explored what people understood by the term “self-harm”, what this looked like in later life, as well as the barriers and enablers for talking about these issues, and who could help, when, where, and how? We did not adopt any definition of self-harm in terms of intent or otherwise. Questions were framed to capture an everyday lay understanding and observations from people's experience. During the interviews, the participants were asked not to mention any identifiable names or places.

The topic guide was piloted with one person (aged 82). The data from the pilot was included in the data analysis as no changes to the topic guide were made following the pilot interview.

All interviews took place remotely due to COVID-19 restrictions. The participants were given the choice of Zoom, WhatsApp, or telephone. Interviews were audio recorded with consent and ranged in duration from 30 to 74 (mean = 53) min.

Approximately one-third of participants wrote spontaneously to various team members by e-mail following the interview stating that they wished to provide further information. These communications appeared to be triggered by not having been used to talking about the topic, with participants realizing that they had since had more to say. Permission was sought to use these further data in the analysis/write up.

### Data analysis

**Figure 1:**
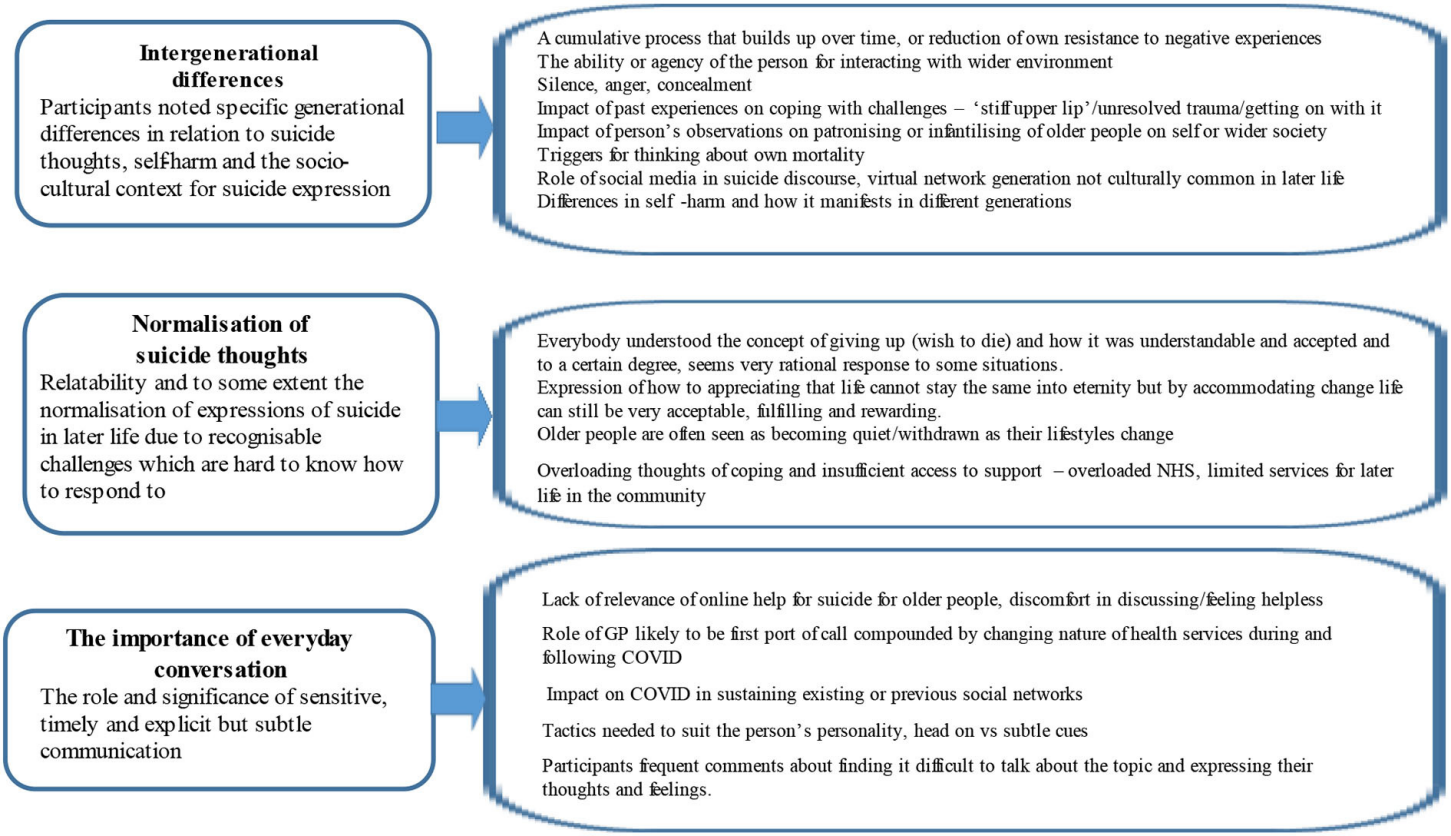
Schematic diagram of qualitative themes.

The interview data were professionally transcribed and anonymized and subject to inductive reflexive thematic analysis (Braun and Clarke, 2012; Clarke and Braun, 2017), which was a theoretically flexible interpretative approach to analysis. A total of two team members (THL and TE) read every transcript separately to familiarize themselves with the interview data and made some initial notes on potential data items of interest, questions, connections between data items, and other preliminary ideas. Then, they manually coded the data, initially by using open coding, noting common phrases and words. Discussion took place after these phases to identify potential categories and subcategories of data ground in the participants' voices. Finally, having gone back to identify linkages across the transcripts and between different categories, further discussion and refinement of these resulted in the identification of three broad themes. The team retained audit trails evidencing decision-making throughout the analytical process. This approach was in line with quality criteria reporting (COREQ; Tong and Sainsbury, 2007) and was undertaken to improve the trustworthiness and credibility of the research process (Shaw et al., 2019). Figure 1 describes the thematic schema from the data with a brief description of the theme and some of the key areas developed from the coding categories.

## Results

Table 1 shows the characteristics of the 15 participants. One-third of the participants were 85 years or older, two-thirds were male, and one-third of the sample identified as lesbian, gay, or bisexual.

**Table 1:** Characteristics of sample (*n* = 15).

**Country of resident in UK**		**Age range**		**Ethnic origin**		**Religion**	
England	11	70–79 yrs	5	Asian Indian	1	Christian	9
Scotland	3	80–84 yrs	5	White English	6	Judaism	2
N. Ireland	1	85–89 yrs	5	White Scottish	3	Protestant	1
		White British	2	Agnostic	3
		White other (American/Jewish)	3	
**Sex**		**Gender identity**		**Sexual identity**		**Disability**	
Male	10	Cisgender	15	Lesbian	1	Yes	5
Female	5			Gay	2	No	10
				Bisexual	2	
				Heterosexual	10	

A total of three themes were identified: (1) intergenerational and socio-cultural differences in suicide expression; (2) the normalization of suicidal thoughts in later life, and (3) the difficulties and importance of discussion (described by some participants as everyday informal conversation), in spaces and relationships where suicidal thoughts are expressed.

### Theme 1: Intergenerational and socio-cultural differences in suicide expression

The first theme captured participants' observations about the specific generational differences in relation to suicidal thoughts, presentation of self-harm, and the socio-cultural context for expressing suicidal thoughts and any behaviors that follow. It was clear that participants viewed suicide expression in older adults to be unique when compared with other age groups.

Firstly, participants discussed the intergenerational differences in the triggers of suicide expression. The participants spoke about how suicidality in younger generations may be more likely to reflect a “crisis situation”, whereas in older adults, this may be more likely to reflect an existential recognition that one's life is “coming to an end”. The participants spoke about how they felt that suicidal thoughts in older adults are more likely to be the consequence of a prolonged or chronic life stressor, rather than an acute response to an acute stress. Commonly discussed examples included chronic pain, disability, and loneliness. As one participant said,

*I think there comes a time for most people that they feel they're coming to the end, that they've done enough, and it's their sort of time. […] Purpose and meaning always comes up for me in life*. (70–74 yrs)

The participants gave many examples of commonly used phrases that older people used to express suicidal thoughts when interacting with their peers. Some were explicit such as “I'm ready to go now,” “I'm tired of life,” or “What's the point anymore?” How and when to pick up on these phrases were discussed by the participants as a crucial but challenging step in helping to identify those at risk for suicide and starting a conversation around these topics. The participants also discussed ambiguity around making the distinction between throwaway comments made to start a discussion and statements of serious intent:

*I do sort of feel in older people, really, that, well there must be two things, there must be those people who feel, now is my time, and I really want to go, and there must be the other people who, it is a cry for help, and they're wanting an opportunity to discuss it*. (70–74 yrs)

Another clear difference related to the specific behaviors that older people would engage in if they sought to harm themselves and how this might not be easily observable when compared with behaviors observed in other age groups is quoted as follows:

*Self-harming, I think, people self-harm in different ways, they don't have to cut themselves, but they do like to get drunk, I would call that a self-harm. It's getting out of what's happening now. I don't know, they might take some tablets that might make them go to sleep for a while and keep doing that. But, self-harming with kids, where they get the razor and cut their arms, and such things like that, yeah, that's a little different*. (80–84 yrs)

An “overt” range of expression of self-harm (e.g., cutting oneself) noted in younger people was contrasted with non-overt, self-neglectful expressions of self-harm in older adults. Here, participants were more likely to include terms such as “giving up”, “shutting the world out,” deliberately not looking after oneself, stopping eating or drinking, refusing to take important medication, or otherwise engaging in unsafe or destructive behaviors, for example, excessive drinking or alcohol or knowingly eating high-sugar foods if the person was diabetic and at risk of developing complications. Whilst these may not lead to suicide directly, these were seen as an expression of suicide. The participants felt that a whilst a direct intent to end one's life might not be evident, the cumulative effect or a lack of care for one's life could nonetheless hasten death. However, as these expressions are often difficult to identify and spot identifying this expression, identifying these “*…would take someone who knows them well and perceives the change in their behavior and attitude”* (80–85 yrs).

Furthermore, generational differences in expressing suicidal thoughts and associated actions were perceived to be influenced by the role of social media in the suicide discourse where the (younger) virtual network generation is active in engaging with information online, exchanging experiences with people they may not personally know. These were noted as being culturally alien for people in later life, not only because of lesser access or familiarity with virtual networking but also due to a cultural influence. Older people were seen as stalwart and more resilient and would be expected to manage or cope without involving others unnecessarily. It was commonly stated that older people might take their feelings and problems to somewhere more familiar and that suicidal thoughts may manifest in different ways.

The experience of stigma was also evident within this theme. Some participants shared their observations of how the patronizing or infantilizing of older people can increase their vulnerability if they were already experiencing suicidal thoughts. One participant particularly noted the vital importance of *how* a professional demonstrated empathy. Again, there were intergenerational differences as “*professionals have never actually experienced being old, they can only say what they think is best for people in later life whereas at the other end, most people have been a teenager themselves*” (75–79 yrs). Another example was given of the random “cruelty in older life” (80–84 yrs). This individual spoke of his own reaction to receiving the standard letter from the U.K. Government Driving Vehicle Licencing Authority sent to all people aged 70 years removing license for driving in all categories of vehicle, except cars. As a recipient, he found the letter arbitrary and “stark” with a message that people his age are “now useless and losing their freedom”. This was described as one of the messages from society to older people that not only is a personal loss of potential independence but signals how people by merit of their chronological age alone are cut off by society.

Many participants also highlighted the importance of past experience of trauma in influencing mental health and suicidal thoughts in later life:

*Secrets are like stones, they weigh you down, and if you… get rid of them, you'll float. But otherwise, you'll drown, and yeah, I mean, people say, oh, I deal with it by hiding it away. Well, no, it's going to come back and bite you. One day, sooner or other, you'll pay the price for that. I think of some of the people that I've worked with through Citizens Advice who've had dreadful experiences in their early years and continuing into adulthood that have not been addressed. It's that late disclosure of stuff that went on earlier, and that can happen right up'til much older people. There's been stuff recently, hasn't there, about the women whose babies were adopted when they're against what they wanted, and they've carried that through forever*. (70–74 yrs)

The references to past trauma from participants, which may be very much seen as taboo, were again described as a generational difference. Older people may not have had access to support, which is more commonly within the reach of younger generations, such as talking therapies, support groups, and through media or social media. This was thought to be more a potential cause of a person becoming silenced, experiencing anger turned in on themselves, and subsequent concealment of suicidal thoughts in later life.

Many of the participants touched on the pandemic by talking about having their wings clipped when time is limited. This led to a need to go out and make the most of opportunities, where possible, post-pandemic because of having glimpsed the potential restrictions that had disproportionately impacted on those in later life and the public response to it. Some participants spoke in detail about the lockdown experiences and the homogenization of older people that had exacerbated any vulnerabilities, and the inevitable overloading thoughts combined with less coping strategies and insufficient access to support. One participant who spent 46 years in an unhappy marriage said,

*he really has destroyed my morale, and with what's gone on in the past 18 months, I don't feel like I want to go on very much further, to be very honest, I don't want to kill myself, but I get to some days where, is this really worth it? And I don't know how many people out there feel like that at my age, but I'm sure there are*. (80–84 yrs)

### Theme 2: Normalization of suicidal thought in later life

This theme concerned the relatability and, to some extent, the normalization of expressions of suicide in later life. Whilst only a few participants shared some of their own experiences of suicidal thoughts, the majority provided many examples of their interactions with peers who experienced suicidal thoughts. Whilst this was experienced as a challenge that they felt very unsure about, the participants generally agreed that suicidal thoughts were understandable and even acceptable in later life. The participants also discussed how, to a certain degree, such thoughts seemed a very rational response to some situations that people faced. For example, as one participant said,

*Giving up, giving in. They're different, but when does one actually lead into the other? And at what point does the idea of keeping on, keeping on, keeping going…which I think many older people have this tremendous capacity to keep going, but does there comes a point at which this seems to be too much of an effort, it seems counterproductive*. (75–79 yrs)

This normalization of suicidal thoughts came from participants' frequent observations of friends and older family members making “off-hand” comments, such as “you don't need me around anymore” or “my time is up”, which they believed to imply that the individual was engaging in suicidal thoughts. Whilst the participants acknowledged that suicidal thoughts are less likely to be recognized in professional interactions between older people and carers or healthcare providers, these thoughts were more commonly recognized in their peer groups such as within friendships and social circles. Indeed, some participants spoke about openly discussing suicide and suicide ideation with their friends:

*The conversation ranges quite widely, and I'm trying to think if we've talked about suicide, and whether we should? And I certainly have a group of friends I know would not be uncomfortable discussing suicide, in the way you and I are now, as another life option, although that might sound a silly thing to say. We're very aware that we have physical problems that have changed our lives, and I think particularly, I'm 75, I think we're noticing very much that our life changes quite dramatically at this age*. (80–84 yrs)

Throughout the interviews, it was apparent that discussions about suicidal thoughts among peer members appeared normalized. Another participant who e-mailed the interviewer post-interview clearly illustrated some of these introspective and prospective considerations of these life course changes and a natural sense of finitude and the inevitabilities of how suicidal thought becomes normalized:

*For me as I have entered each new decade there have been changes. Looking back to my 20's, 30's, 40's, I had boundless energy, and it was easy to follow my dreams. Jobs were plentiful, opportunities there for the taking, no internet to absorb time or influence one. Lifelong friends were established, and discussions focused on planning experiences together, having fun and traveling to explore the world. It felt as if you could achieve whatever you chose to follow. Entering my 7th decade felt like a real marker in terms of thought processes, and discussions with friends of similar age turn to the inevitable time limit of years ahead of me. I no longer see time stretching out before me but have a realization that years are limited which focuses my mind on how to use the time I have left, and I have concentrated on living in the moment as much as I can. There is a sense of loss, grief and change with this decade and with that some feelings of depression and giving up*. (70–74 yrs)

This expression of appreciation that life cannot stay the same but by accommodating change, one's expectations would have to change, was a theme that ran throughout several interviews. Whilst there was some degree of othering, the participants were easily able to be in the shoes of their peers, and this relatability to experiencing suicidal thoughts was very much present. These findings are in line with the life review literature that has highlighted that it is the important role of personal meaning that is attributed to past events that are relevant to how we regulate our identity and wellbeing (e.g., Butler, 1963; Bluck, 1998; Westerhof, 2014; Adler et al., 2015).

A 77-year-old man referred to how losing a loved one or friend was very common in later life and could be more devastating if a person had not developed a wider range of friends, diverse interests, and hobbies separate to their relationship with a partner. He spoke of having experienced the death of his wife and how focusing his energy on following his own interests had helped him through the grieving process (75–79 yrs).

### Theme 3: The difficulties and importance of discussion

Despite the apparent normalization of suicidal thoughts, many participants made frequent comments about finding it difficult to talk about suicide and to express their thoughts and feelings surrounding suicide, especially in a formal setting. The key message within this theme was the importance of both being able to adapt to the individuals' personality and being able to pick up on subtle cues when discussing suicide-related thoughts and behaviors. The participants particularly highlighted the importance of everyday informal talk. These allowed the discussant both to acknowledge the personal as an individual and to tread the fine line between their right to autonomy and their right to support. These difficulties and importance of discussing suicidal thoughts with older adults appeared related to generational issues about discussing “feelings” and “emotions”, due to “stiff upper lip upbringings”. For example,

*Well, I suppose it can be quite difficult for older people who are not used to sharing their emotions or talking about their emotions. They find that very difficult. They've been brought up to keep a stiff upper lip and not to think too much about themselves really. Well, a lot of them don't even understand their feelings*. (86–89 yrs)

*I think with us older generation, we came up just after the war, like 70s above, we're what we call the war babies. And the people above us are the people that lived through the war and whatever went on. And in those days, you got on with it. […] you got on with life, you had to*. (80–84 yrs)

The participants spoke about many older people feeling “stigma” and “shame” when discussing mental health, and how asking for help is seen as a “sign of weakness”. Those participants who had experienced mental health problems said how stigmatized they were within their own generation even though mental health was now commonly talked about in the media. Despite this, the importance of tackling these difficult points of discussion was highlighted throughout the dataset. Just because some older people may find it difficult to discuss these topics, it does not mean that they do not want to seek help:

*The younger ones can be prepared to reach out. Again, they've not had the upbringing where it's a sign of weakness to reach out. That is the important one, with the caveat being that, again, the older person can still have the feelings, emotional feelings, deep down that they do want help*. (75–79 yrs)

The participants referred to a lack of relevance of online help for suicidality for older people, and the discomfort in discussing/feeling helpless. Most of them cited the role of GPs who were likely to be first port of call compounded by the changing nature of health services during and following COVID. Whilst the GP was commonly cited as a source of help-seeking across the interview, there were some reservations about the limitations of places where people could go:

*No, I don't actually, because in the first place, being absolutely cynical, I think they'd find out after a lifetime of GPs that, really, they're not going to get much help, are they, from the National Health Service side of things, or there is a very limited help available. And what they perceive that they need is perhaps not a medical…they don't consider themselves as being medically needing help, so they wouldn't sort of say to their GP. Because you know, if you go to your GP and you say, I'm depressed, well, she'd just write you out a prescription for tranquilizers, won't she? But she won't go into deeper detail, but why are you depressed; because the reason you're depressed and you're lonely is because you haven't got enough money, where you live is perhaps inadequate, and you don't have enough social interaction with other people. And your GP can't do anything about that, can she?* (80–84 yrs)

The participants spoke about using “person-centred” and easy-to-understand language to initiate these conversations. Prompts such as “I've noticed that you seem quite low, have I got that right?” and “Come on a walk with me, I want to hear how you have been recently” were identified as potentially useful ways to broach these potentially challenging topics. Friends, family members, and carers/staff members working in sheltered housing schemes were all identified as suitable people to initiate such discussions. For instance,

*I think, probably they (older people) are not so much used to talking about their feelings… maybe it is more dependent on those who are looking after them to pick up on it, and to raise the question. […] I mean, I think with staff, one has to look at who gives up? I think sometimes there's two things going on. Sometimes it's the staff that don't want to listen and don't want to hear, and don't want to observe what's going on, and it's the old person that's crying out to be heard and listened to. So, I think their staff need the support, to know how to talk to someone, what to say at that level maybe, what to say, what language to use, how to engage the person? And to know that it's important to do so, and you can't just be happy all the time*. (70–74 yrs)

This individual focused on some of the dynamics between older people and their carers and how both experienced discomfort and challenges in giving and responding to cues. He spoke about the importance of being able to stay in the moment when these cues come up and the potential for thwarting any fruitful dialogue:

*I can remember my dad saying, oh, you know, he was looking really bad, he'd lost a lot of function. And he said, oh, what a life. And obviously clearly depressed, but in a way, I didn't want to hear it. But I think for me at that point, it's several years ago now, but at that point I had a sense of doing, I had to do. And I think staff and families need to have a sense of being, you know, that you have a conversation that's about being with the person, hearing, listening, you know, exploring. It's not necessarily about leaping off and doing things, or changing. Or trying to change the situation I mean*. (70–74 yrs)

Many participants described that responding to suicidal thoughts in later life was very much part of their experience and something they felt they understood and were able to empathize with. However, they also acknowledged that it remained challenging for them to know how and where to provide support. Some participants specifically noted the economic disparities and weaknesses in healthcare and other support systems since the COVID-19 pandemic. These participants expressed pessimism about the ongoing tensions in prioritizing resources across different generational groups and acknowledged that this is a difficult issue for those in decision-making roles.

## Discussion

This exploratory study of suicidal thoughts in later life, from the perspective of lay older people, provides further insights into how public health and suicide prevention efforts can effectively support older people who are dealing with these situations. With the growing emphasis in public policy on positive or successful aging, stigmatized or taboo topics such as suicidal thoughts are often ignored, or discussed to a lesser degree (Clarke et al., 2012; Hafford-Letchfield et al., 2022a). Within the three themes identified here, the notion of suicidal thoughts and their passive expression appear to be common among aging peer groups and also, to some degree, normalized. As an enabling factor, it may be that this awareness of finitude (Marshall, 1986) could be better acknowledged and discussed, given that those who express a desire to talk about or discuss end-of-life plans are often silenced and/or dismissed as being overly morbid (Kjølseth et al., 2010; Hafford-Letchfield et al., 2022a).

### Improving opportunities for disclosure and authentic discussion

In terms of unique factors associated with suicidal thoughts in later life that could aid recognition, one of the key findings of this work relates to the informal and “off-hand” comments used by older people to express their feelings and engagement with suicidal thoughts. For example, the participants spoke about such thoughts using terms such as being “fed up” or “tired” with life or making statements such as “what's the point anymore?” In line with earlier work (Frey et al., 2018; Calear, 2019), the stigma surrounding mental health and suicide-related topics in later life appeared to mean that these individuals felt more comfortable discussing these topics in colloquial ways through informal (e.g., friends), rather than formal, networks (e.g., GPs or other healthcare providers). Older people progressing from more passive (e.g., suicidal thoughts or wishes to die) to active suicidal expressions (e.g., neglect or self-harm with intent) are also more likely to engage in implicit, rather than explicit, expressions/behaviors (Wand and McKay, 2022). For example, these individuals may begin deliberately not looking after themselves, stopping eating or drinking, refusing to take important medication, or otherwise engaging in unsafe or destructive behaviors, for example, excessive drinking and eating high-sugar foods if the person was diabetic, or not seeking help for serious medical conditions. However, as these expressions are often difficult to identify, particularly where individuals are presenting with complex health conditions and multi-morbidities, participants reported that the recognition of these requires someone who knows the individual well so that subtle changes in their behavior and attitude can be identified.

Further complexity involving ethical considerations may occur, for example, where there are advanced directives and/or mental capacity in death-hastening behavior (Wax et al., 2018; Trowse, 2020; Hafford-Letchfield et al., 2022b). Wand et al. (2018) suggested that self-neglect should be conceptualized as a defensive behavior that has maladaptive outcomes and can also be associated with attempts to regain control over personal freedom and/or living arrangements, or in response to threats to self-identity. Instances where an older person seeks to hasten death such as those that commonly occur in care homes (Hafford-Letchfield et al., 2020) require skilled, detailed assessment to respond to risks alongside improved training and support for paid carers to achieve a more holistic strategy, which capitalizes on significant relationships within a wider context. This raises an important question: who is best placed therefore to identify and support older people experiencing suicide-related thoughts and behavior?

### The wider impact of ageism

The participants in this study identified everyday ageism (ageism that is embedded, is taken-for-granted, and informs day-to-day interactions and experiences of people in later life) to be one of the key barriers to obtaining help for suicidal thoughts (Angus and Reeve, 2006; Bodner et al., 2018; Voelkner and Caskie, 2022). More research is needed to investigate how everyday ageism may be associated with health disparities within the older adult population, and specifically what helps to moderate associations between everyday ageism and suicidal thoughts. Allen et al. (2022a) found that the odds of negative health outcomes increased significantly in individuals experiencing everyday ageism, with this affecting health outcomes *via* multiple pathways, including hampering the quality of older adults' interactions with health clinicians. Everyday ageism, defined as “brief verbal, nonverbal, and environmental indignities that convey hostility, a lack of value, or narrow stereotypes of older adults” (Allen et al., 2022b), may be subtle and not intentionally discriminatory but includes being patronized (Hehman and Bugental, 2015) or subject to communication, which disregards wellbeing and equal access to support and services with other age groups. The taboo of talking about suicide and silence, sometimes by taking a protective stance, was illustrated in some of the challenges the team faced in engaging organizations that were reluctant to circulate details of recruitment to this research study as they genuinely believed that engaging in conversations about the issues would pose some sort of risk to older people in itself.

There is some consensus in the literature on self-perceptions of age and value held by older people that contributes toward internalized ageism (Kydd and Fleming, 2015; Diehl et al., 2021). These self-perceptions were evident in the “throwaway” comments our participants referred to in their peer networks (e.g., “I'm ready to go now” or “my time is up”). Such statements highlight the further need for compassion and high-quality care that pays attention to the nuancing or tailoring of mainstream suicide prevention and mental health therapies for people in later life (Hafford-Letchfield et al., 2022a,b). Validated screening tools used in suicidality with older people tend not to address these wider issues (Gleeson et al., 2022).

In the context of these findings, relationship and language are absolutely key in enabling the discussion of suicidal thoughts. Studies have shown that many older people are in contact with health services shortly before the suicide, but without the subject of suicidality being taken up during the consultation (Harwood et al., 2000; Luoma et al., 2002). This supports the argument that training of general practitioners in how to recognize and treat depression or suicidality is a vital part of multilevel suicide prevention strategies (e.g., van der Feltz-Cornelis et al., 2011; Claassen et al., 2014). Our findings highlight the importance of professionals learning to identify implicit/passive suicidal expressions in later life and being willing to address it. There is significant variability in suicidal thoughts that the informal “natter” does really matter in providing ongoing support, as opposed to a one-time screening where older people may be either unwilling or unable to reveal active suicide thinking when responding to direct questions about suicide (Hawton et al., 2022; Rudd and Bryan, 2022). Internal and external influencing factors (e.g., trauma, stigma, and loss of autonomy) may result in fleeting non-specific thoughts with no significant (active) wish to die or reflect chronic yet passive suicidal thoughts that do not elevate risk of actual suicide. Further studies comparing bereavement experiences of those bereaved by suicide in later life with other traumatic bereavements and losses will also help understand the individual experiences and pathways within suicide research to help inform and enrich assessments and interventions in aging care (Hybholt et al., 2020; Hafford-Letchfield et al., 2022b).

Key access points to life-sustaining conversations may be important for those using home- and community-based services as these individuals often face additional barriers to accessing mental healthcare (Qiu et al., 2010; Wyman and Shiovitz-Ezra, 2018). There appears to be a relatively unexplored potential for peer support among older people, given how our participants recognized the common ground shared and acknowledged how isolating it can be for people who are not able to access professional services or feel stigmatized in doing so. There is a level of understanding and relatability with peers that could form a valuable source of support to help people talk more openly about it. Indeed, the participants in the present study revealed regularly discussing these topics with their friends and peers. Some studies in suicide research have found that people may perceive their end of life positively and are open to discussing the issues (Van Der Geest, 2002; Gilleard, 2022). Innovative approaches to suicide prevention that bring care into the home have been recommended (Salvatore, 2015; Westcott et al., 2022). This could involve training carers or volunteers who interact on a regular basis with socially isolated older people in the role for what Westcott et al. (2022) terms “natural helpers”. They referred to those who have access to people at risk of suicide by virtue of a role (e.g., occupation) and personal characteristics (i.e., empathy) that equip them to connect with those people. Furthermore, research on any outcomes following such low-level interventions would be useful to inform suicide prevention strategies.

## Conclusion

This study brings the unique perspective of lay people with respect to suicidal thoughts and ideation in later life, describing their views and experiences of suicidal thoughts and the everyday interactions that might bring these to the fore and enable more open discussion. A better understanding of the transition from thinking about suicide to engaging in suicidal behavior is critical. Current developments in idea-to-action frameworks will be important in developing theories to inform interventions and postventions for people in later life. This distinction between idea to action is important as the majority of individuals who experience suicidal ideation do not necessarily make the progression to suicide attempt (Klonsky and Saffer, 2018). Additionally, frequently identified risk factors for suicidal ideation, such as depression, trauma, and hopelessness, do not differentiate between suicide ideation and suicide (Rudd, 2021).

The present findings highlight the importance for professionals and providers to be more open to discuss topics related to suicide in later life, acknowledging and communicating a willingness to talk about these matters in an informal setting. Training those staff with improved awareness at the everyday level where discussion takes place, such as personal care or the persons' own home environment, supported by improved signposting and assessment is another area for development. Given the elevated risk of suicide in later life and the conceptual frameworks available (Hafford-Letchfield et al., 2022b), innovative approaches to suicide prevention are important to a public health approach to suicide that involves people in later life as stakeholders.

## Study limitations

Some of the findings of this study reflected the U.K.-specific context. There are context-specific discourses about older people's access to healthcare, which may be different than other countries with different health and care systems. There is no euthanasia legislation in the United Kingdom, which may influence debates. This study was based on a small sample, and the challenges in recruiting people from more diverse backgrounds to discuss this topic is acknowledged.

## Data availability statement

The raw data supporting the conclusion of this article can be made available by direct request to the corresponding author.

## Ethics statement

The studies involving human participants were reviewed and approved by University of Strathclyde Central Ethics Committee. The patients/participants provided their written informed consent to participate in this study (Ref: UEC21/67).

## Author contributions

TH-L conceptualized and designed the study and secured funding. JH and TH-L obtained ethical approval. JH coordinated participant recruitment and the majority of qualitative interviews with input from TH-L, TE, NC, PW, and HG. TH-L and TE analyzed the data. TH-L drafted the manuscript with substantial input from TE and HG. JH, NC, SR, and JG edited the manuscript. PW, MQ, and SM contributed to the wider research team discussions and tasks. All authors contributed to the article and approved the submitted version.

## Funding

This project was supported by the University of Strathclyde New Professors Fund.

## Conflict of interest

The authors declare that the research was conducted in the absence of any commercial or financial relationships that could be construed as a potential conflict of interest.

## Publisher's note

All claims expressed in this article are solely those of the authors and do not necessarily represent those of their affiliated organizations, or those of the publisher, the editors and the reviewers. Any product that may be evaluated in this article, or claim that may be made by its manufacturer, is not guaranteed or endorsed by the publisher.
